# Sexual violence among female survivors in Goma, in the Democratic Republic of the Congo: epidemiology, clinical features, and circumstances of occurrence

**DOI:** 10.1038/s41598-024-65412-7

**Published:** 2024-06-27

**Authors:** Yannick Nkiambi Kiakuvue, Furaha Siwatula Kanyere, Doris Mwila Mukubu, Bienvenu Mukuku Ruhindiza, Olivier Mukuku

**Affiliations:** 1 Faculty of Medicine, University of Lubumbashi, Lubumbashi, Democratic Republic of the Congo; 2 Faculty of Medicine, University of Goma, Goma, Democratic Republic of the Congo; 3 Faculty of Medicine, University of Ngozi, Ngozi, Burundi; 4 Institut Supérieur Des Techniques Médicales, Lubumbashi, Democratic Republic of the Congo

**Keywords:** Sexual violence, Female survivors, War, Conflict, Democratic Republic of the Congo, Health care, Public health, Epidemiology

## Abstract

Sexual violence (SV) is a major public health issue in Goma, Democratic Republic of the Congo, especially in the eastern part of the country where women have been victims of SV for many years. The objective of this study is to provide an overview of the survivor and perpetrator characteristics, as well as the circumstances surrounding SV incidents in Goma. We conducted a retrospective, descriptive cross-sectional study using data from all SV survivors who sought medical care at four hospitals in Goma from January 2019 to December 2020. The analysis of the data was carried out using STATA 16 software. A total of 700 women sought medical attention for SV in the four hospitals. The survivors’ age range was 12–67 years with a mean age of 31.7 ± 14.6 years. Women aged 20–29 years were the most affected (28%). The majority of SV survivors experienced their first assault (88.29%) and sought medical attention within 72 h (60.6%). The assaults occurred mostly outside the SV survivors’ homes under armed threat (84.29%), predominantly by men in civilian clothes (61.43%) compared to men in military uniform (38.57%). More than half of the survivors were assaulted by a stranger (64.71%), and of those, more than half were committed by a single perpetrator (57.29%). The findings underscore the urgent need to address this pervasive issue, emphasizing the necessity of targeted interventions to protect survivors and prevent future incidents. The circumstances surrounding these assaults, such as the prevalence of armed threats and attacks outside survivors’ homes, highlight the complex challenges in combating SV in this region.

## Introduction

Sexual violence (SV) encompasses actions such as verbal harassment, non-consensual penetration, and various forms of coercion that range from societal pressure and intimidation to the use of physical force. SV, including rape, constitutes a violation of human rights with significant health implications^1^. Recent global prevalence data indicate that an estimated 35% of women worldwide have experienced intimate partner violence or non-partner SV at some point in their lives^2^.

In 2018, it was estimated that one in three women, equivalent to an average of 736 million to 852 million women aged 15 years or older, had experienced at least one incident of intimate partner violence, non-partner SV, or both during their lifetime^3^.

A systematic review conducted from 2010 to 2018 revealed that central sub-Saharan Africa (32%) and Oceania (29%) had the highest lifetime prevalence of intimate partner violence and physical or SV within the past year among women aged 15–49 years^3^. SV is a major public health issue in Goma, in the Democratic Republic of the Congo (DRC), especially in the eastern part of the country where women have been victims of SV for many years. In the DRC, a study conducted in 2007 reported that approximately 1.69–1.80 million women aged 15–29 years reported having been sexually assaulted in their lifetime, with 223,262 of them from the province of North Kivu alone. The study also indicated that North-Kivu province, followed by Province Orientale and Équateur, had the highest rate of lifetime SV^4^. A 2010 study in the North and South Kivu provinces reported that 39.7% of women in the eastern DRC had experienced SV in their lifetime^5^. From 2013 to 2017, a study conducted in the city of Goma revealed that SV predominantly affected females under the age of 18, with a mean age of 16.5 years. Notably, half of the perpetrators were civilian individuals who were acquainted with the survivors, 12% of the survivors tested positive for pregnancy, and an additional 43% received emergency contraception^6^. SV has also been recognized as a tactic employed in the context of armed conflict in the eastern region of the DRC^7^. However, Baaz and Stern^8^ suggested that SV in the DRC cannot simply be explained as an unavoidable aspect of war or merely as a “weapon of war”. Instead, it reflects complex power dynamics and deeply ingrained cultural narratives. Soldiers described SV as resulting from masculine heterosexuality and the belief that men have sexual needs that must be met, sometimes through force. They also linked rape to frustration caused by poverty, neglect, and the chaotic environment of wartime. This frustration manifests in acts of violence against civilians, including rape, which are viewed as a temporary assertion of masculinity. However, this act also represents their failure to achieve true masculinity, as it deviates from the “real” notions of masculinity they aspire to Baaz and Stern^8^. Another study examining the phenomenon of SV among combatants revealed a profound disconnect between the ideals and actions of soldiers. Despite strongly believing that SV is a serious crime and an immoral act that should be avoided, soldiers sometimes perpetrate such violence systematically. This contradiction highlights a tension between their professed commitment to good soldiering ideals and their actual behavior. Combatants reported a desire to protect women and men in armed conflicts; however, the challenging contexts in which they operate often lead them to act violently against the very civilians they are supposed to safeguard^9^. A study conducted by Cohen and Nordås^10^ suggested that the prevalence of SV varies dramatically by perpetrator group, indicating that while SV is common, it is not ubiquitous. State militaries are more likely to be reported as perpetrators of sexual violence than either rebel groups or militias. Furthermore, SV continues into the post-conflict period, sometimes at very high levels^10^.

Despite receiving widespread media attention, SV in the DRC has not received enough attention in the medical literature. More research is necessary, especially to understand the significance of militarized SV in comparison to civilian rape and how it affects vulnerable populations. The impact of SV on health can manifest in various ways, including immediate and severe effects, prolonged and chronic consequences, and, in some cases, even leading to fatality, such as femicide. These effects encompass acute or immediate physical injuries, female genital mutilation, unintended and unwanted pregnancies, abortion/unsafe abortion, and an increased risk of contracting sexually transmitted diseases, including HIV^11–13^. The interest given to this subject is motivated by the high frequency and consequences of SV endured by women. The objective of this study is to delve into the characteristics and experiences of female survivors of SV in Goma, identifying the perpetrators, and dissecting the circumstances under which SV occurs. By honing in on this locale, the research aspires to deepen the collective understanding of the magnitude and essence of SV, pinpoint the risk factors associated with dire health outcomes, and critically appraise the effectiveness of current interventions. This study endeavors to provide a solid empirical foundation upon which policies and interventions can be constructed or refined. The ultimate goal is to decrease the prevalence of SV and to elevate the health and quality of life for survivors in the DRC, as well as in other areas plagued by conflict and pervasive SV. Through meticulous investigation and analysis, this research seeks to contribute meaningful insights to the global discourse on SV, offering tangible solutions to mitigate this profound public health and human rights crisis.

## Materials and methods

A retrospective descriptive cross-sectional study was conducted, focusing on women who sought care at four selected healthcare facilities in the city of Goma from January 1, 2019, to December 31, 2020, and who have experienced SV. The study population is limited to the four facilities that provide support to women who are survivors of SV. Only cases of SV were included in the study, and cases of physical violence were excluded.

Goma is a city situated in the eastern part of the DRC, at an altitude of around 1500 m in the Rift Valley. Serving as the capital of the North Kivu Province, it covers an area of 66.45 km^2^ characterized by undulating relief and volcanic rocks near Mount Nyiragongo. Positioned at 29° 14′ East longitude and 1° 45′ South latitude, Goma borders the Nyiragongo Territory to the north, Lake Kivu to the south, the Republic of Rwanda to the east, and the Masisi Territory to the west.

With a high population density of over 2333 inhabitants per km^2^, the city consists of two municipalities (Goma and Karisimbi), two health zones (Goma and Karisimbi), along with 10 health areas in Goma and 19 in Karisimbi. Goma is divided into administrative districts within its two urban municipalities. The healthcare facilities providing support for survivors of SV that we examined are religiously affiliated institutions co-managed with the Congolese government and backed by the MSF/France Partner and *HEAL Africa* through their organization *Tushinde Ujeuri*.

To form our sample, we contacted the health facilities that provide care for survivors of SV in Goma. Although there were 10 such health facilities, only 4 of them gave permission to use their data. We obtained access to the data by retrospectively collecting them from medical records of patients in the four healthcare facilities. The questionnaire administered compiled demographic details such as age, history of SV, physical disability (present or absent), alongside information pertaining to the sexual assault incident, encompassing details on the site (home or elsewhere), the nature (armed or unarmed), the type (exclusively SV or SV with physical violence), condom use by the perpetrator during the assault (yes or no), the perpetrator’s status (civilian or military/police), the relationship between the patient and the perpetrator, the number of perpetrators, assault reported to the police (yes or no), whether the perpetrator was a minor or an adult, their civilian or military status, the location and date of the assault. The patient’s medical records documented any presenting complaints upon hospital arrival, time between assault and arrival at the hospital, physical examination findings (including presence of lesions, bruises, redness, and semen), and results from pertinent tests such as those for pregnancy and HIV. Moreover, details of prophylactic treatments provided to the patient were delineated, encompassing anti-retroviral medications for HIV prevention, hepatitis B vaccination, antibiotics for sexually transmitted infection (STI) prevention, and emergency contraception for pregnancy prevention. All data were initially recorded on paper forms and subsequently entered into a Microsoft Excel spreadsheet.

A case of SV was defined as per the World Health Organization’s definition, which states that SV includes any sexual act, attempt to obtain a sexual act, comment or advances of a sexual nature, or acts directed against a person’s sexuality through coercion, committed by any person regardless of their relationship to the victims, in any setting, including but not limited to the home and workplace^1^.

The study focused on a sample of 700 women who were survivors of SV. The collected data were entered, encoded, and analyzed using STATA 16 software. Tables presenting the variables were created using Microsoft Excel 2019 software. Proportions, means, and standard deviations were calculated for the analysis and interpretation of the data.

The study ensured anonymity during data processing by consulting patient records. Before data abstraction, strict measures were taken to de-identify and anonymize the data, ensuring that no personal health information could be linked to individual patients for analysis or writing purposes. Authorization for data collection was obtained from the Chief Medical Officers of the Health Zones of Goma and Karisimbi, as well as from the authorities responsible for the healthcare facilities surveyed. The study received approval from the Medical Ethics Committee of the University of Goma (Approval No.: UNIGOM/CEM/012/2021), and all methods were conducted in accordance with relevant ethical guidelines and regulations. According to the Guidelines for the Ethical Review of Research Involving Human Subjects in the DRC, since the study involves retrospective analysis of existing data collected as part of routine care and there was no contact with patients, individual informed consent was not required^14^. Prior to data abstraction from the chart record, the data were de-identified and anonymized. As a result, no identifying personal health information could be linked to the patient data for analysis or writing purposes.

### Ethics approval and consent to participate

As the study involved a retrospective secondary analysis of existing data collected as part of routine care and there was no contact with patients, individual informed consent was not required, in accordance with the guidelines for the Ethical Review of Research Involving Human Subjects in the DRC. It was a retrospective case record study.

The study received approval from the Medical Ethics Committee of the University of Goma (Approval No.: UNIGOM/CEM/012/2021). Patient data were de-identified and anonymized during data abstraction from the chart record to ensure that no identifying personal health information could be linked to the patient data for analysis or writing purposes.

## Results

During the study period, 700 cases of SV were recorded in the four selected hospitals in the city of Goma. Table 1 shows the distribution of SV survivors according to the local area where the SV occurred. It was observed that the municipality of Karisimbi recorded 551 cases (78.7%) and the municipality of Goma recorded 149 cases (21.3%). The local area with the highest number of SV cases was Majengo, with 129 cases (18.4%).

**Table 1:** Distribution of SV survivors according to the local area where the SV occurred.

Area		Number (N = 700)	Percentage
Municipality	Local area		
Goma	Katindo	61	8.7
	Kyeshero	44	6.2
	Mapendo	43	6.1
	Les volcans	1	0.1
Kasirimbi	Majengo	129	18.4
	Ndosho	68	9.7
	Mugunga	64	9.1
	Bujovu	61	8.7
	Murara	47	6.7
	Kasika	39	5.7
	Mabanga Nord	39	5.7
	Katoyi	36	5.1
	Kahembe	35	5.0
	Virunga	25	3.6
	Mabanga Sud	8	1.1

The study population comprised women aged 12–67 years, with a mean age of 31.7 ± 14.6 years (median 28 years). Among these cases, 28.6% were women aged 20–29 years old. Additionally, the study found that only 15.7% (111 cases) of the survivors reported being assaulted at their own homes, with most assaults involving the use of weapons (435 cases, 60.7%). The assailants predominantly engaged in vaginal coitus in 100% of cases, followed by touching in 7.2% of cases. Only 11.7% of the survivors had experienced prior assaults, with 401 (57.3%) of them reporting being assaulted only once. In less than 1% of cases (6 cases, 0.9%), the perpetrator used a condom. In the majority of instances, the perpetrator was a stranger (453 cases, 64.7%), and in 61.4% of cases (430 cases), the perpetrator was identified as a military or police member. 8.1% of the cases involved survivors with a physical disability (Table 2).

**Table 2:** Characteristics of sexual violence survivors, characteristics of sexual violence perpetrators, and circumstances of the assault.

Variable	Number (N = 700)	Percentage
Age		
< 20 years	169	24.1
20–29 years	200	28.6
30–39 years	148	21.1
40–49 years	71	10.1
50–59 years	77	11.0
60 years and over	35	5.0
History of SV		
Yes	82	11.7
No	618	88.3
Physical disability		
Present	57	8.1
Absent	643	91.9
Site of the assault		
At home	111	15.7
Elsewhere	589	84.3
Nature of the assault		
Armed	435	60.7
Unarmed	275	39.3
Type of the assault		
Exclusively sexual violence	691	98.7
Sexual violence with physical violence	9	1.3
Condom use by the perpetrator during the assault		
Yes	6	0.9
No	694	99.1
Assault reported to the police		
Yes	547	78.1
No	153	21.9
Relation between the perpetrator and the survivor		
Stranger	453	64.7
Person in a position of power (employer, teacher)	188	26.9
Victim’s ex-partner	36	5.1
Family member	20	2.9
Neighbor	3	0.4
Number of perpetrators		
1	401	57.3
2–3	269	38.4
> 3	30	4.3
Status of the perpetrator		
Civilian	430	61.4
Military/police	270	38.6

Of the 700 SV survivors, only 16.1% (113 cases) sought medical consultation for genital hemorrhage, and 0.7% (5 cases) presented with mutism. The majority of the survivors (424 cases, 60.6%) sought post-sexual assault care at one of the four hospitals within 72 h. Among these, 8.1% (57 cases) tested positive for HIV. Pregnancy tests were conducted in 76.3% of the cases, with only 6.4% (45 cases) yielding positive results. Moreover, over half of the SV survivors (388 cases, 55.4%) received contraception treatment, and 91.1% (638 cases) were offered antiretroviral prophylactic medication (post-exposure prophylaxis). Additionally, 98.9% of the SV survivors received antitetanic vaccination, and 93% of them also received hepatitis B vaccination (Table 3).

**Table 3:** Symptoms and sexual violence case management.

Variable	Number (N = 700)	Percentage
Severe genital hematoma		
Present	113	16.1
Absent	587	83.9
Mutism		
Present	5	0.7
Absent	695	99.3
Time between assault and arrival at the hospital		
< 72 h	424	60.6
72–120 h	240	34.3
> 120 h	36	5.1
HIV testing		
Positive	57	8.1
Negative	587	83.9
Not done	56	8.0
Pregnancy testing		
Positive	45	6.4
Negative	489	69.9
Not done	166	23.7
Contraception medication		
Yes	388	55.4
No	312	44.6
Post-exposure prophylaxis against HIV		
Yes	638	91.1
No	62	8.9
Antitetanic vaccination		
Yes	692	98.9
No	8	1.1
Hepatitis B vaccination		
Yes	651	93.0
No	49	7.0

## Discussion

The present study conducted in Goma, the bustling urban capital of North Kivu province in the DRC, has unveiled a series of critical findings that paint a disturbing picture of SV against women. In the Eastern DRC, SV against women is a devastating reality, exacerbated by persistent armed conflict. Such assaults, commonly used as weapons of war, leave deep physical and psychological scars on the victims. Women are especially vulnerable during times of conflict, with armed groups exploiting instability to perpetrate heinous acts with impunity. Displaced women, living in overcrowded camps with no security, are particularly susceptible to such violence. Unfortunately, the existing legal framework often fails to adequately protect women’s rights due to deficient institutions and a lack of resources^15^. Persistent impunity for perpetrators further discourages survivors from reporting assaults for fear of reprisals. It is imperative to strengthen the judicial system, raise awareness in society, and provide appropriate psychosocial support for victims to combat such violence.

A total of 700 women recorded as victims of SV during the study period is just the tip of the iceberg. This alarming statistic underestimates the reality, as many women, despite the atrocities they have suffered, choose not to report the assaults. A number of factors contribute to this painful silence, including fear of reprisals from the aggressor, fear of further violence, shame and stigmatization within the community, and lack of information about specialized medical facilities. It is crucial to understand that this sad reality is mainly concentrated in urban areas, which does not fully reflect the scale of SV in North Kivu province. Rural areas, where armed groups are particularly active, remain major hotspots of such abuse, often escaping official statistics^6^. To combat this violence effectively, it is imperative not only to promote the reporting of existing cases but also to raise awareness among communities, particularly in rural areas, of the devastating consequences of these acts. This should be accompanied by an expansion of information and medical support services, in order to break the vicious circle of silence that surrounds these tragedies.

This study reveals several significant findings. Firstly, the majority of SV survivors were women aged between 20 and 29 years old, with a mean age of 31.7 ± 14.6 years. Secondly, a substantial proportion of SV survivors sought post-sexual assault care within 72 h and received HIV testing and pregnancy examinations. Additionally, over 50% of the SV survivors received contraception treatment, and the vast majority (91.1%) were offered antiretroviral prophylactic medication. Thirdly, the majority of assaults occurred outside the SV survivors’ homes, frequently involving the use of weapons, predominantly by men in civilian clothes (61.43%) compared to men in military uniform (38.57%). More than half of the survivors were assaulted by a stranger (64.71%), and of those, more than half were committed by a single perpetrator (57.29%).

The present study found a mean age of 31.7 ± 14.6 years among the participants. These findings differ from those reported by Paluku et al.^6^, who reported a mean age of 16.5 years. These results contrast with those found by Kandolo et al.^16^ in Lubumbashi (in the DRC), where the age of the victims varied between 2 and 34 years, with a mean age of 13 years. The age group most affected was between 14 and 17-year-olds (62.93%). SV perpetrators were predominantly male (100%), civilian (98%), and adult (86.2%); only 19.18% of SV cases were reported to judicial authorities^16^. A similar observation of the young age of SV survivors was made by Nguessan et al.^17^ in Conakry, Faye Diemé et al.^18^, and Adama-Hondégla et al.^19^ in Togo. These authors attribute the high frequency of SV among children and adolescents to the vulnerability of this population, as they are not yet discerning. Several factors may contribute to this disparity, including variations in the study duration and the inclusion of four hospitals in our study compared to their focus on a single hospital. Additionally, Paluku et al.^6^ included male SV survivors in their analysis, which could also contribute to the differences observed.

The majority of survivors (60.6%) sought medical attention within 72 h, emphasizing the significance of providing timely support and care for survivors. Our findings align with those of Paluku et al.^6^, who reported a slightly lower percentage of survivors seeking medical attention within the recommended 72-h window (57%). The increase in the proportion of survivors seeking medical attention within the recommended timeframe may be attributed to community-level awareness campaigns that highlight the importance of seeking early care for emergency contraception, HIV post-exposure prophylaxis, and antibiotic prophylaxis. In all cases of SV, vaginal coitus was the preferred sexual act by the assailants, followed by touching at 7.2%. This finding is consistent with those of Nguessan et al.^17^ and Faye Diemé et al.^18^, who reported 73.7% and 93% vaginal coitus respectively. Buambo et al.^20^ found vaginal penetration in 80.4% of cases, followed by sexual touching in 12.7% and fellation in 6.9%.

We found that in most cases, there was only one perpetrator (57.3%). Our findings are similar to those of Balde et al.^21^ in Conakry and Amenu and Hiko^22^ in Ethiopia. The primary perpetrators were men in civilian clothes, which is consistent with the findings of Paluku et al.^6^ in Goma. These results demonstrate that SV is not only a weapon of war in the region but also a public health concern in the daily lives of Congolese unrelated to conflict.

This study found that 64.71% of the sexual aggressors had no relationship with their victims and were not known to their victims. Furthermore, we found that the majority of SV survivors were assaulted by strangers, and these assaults predominantly occurred outside of the SV survivors’ homes. Buambo et al.^20^ in the Republic of Congo and Bajos et al.^23^ in France reported 57.90% and 91.20%, respectively, of unknown sexual aggressors. Contrary to what was reported in the city of Lubumbashi (in the DRC) by Kandolo et al.^16^, the majority of SV were committed by someone known to the victim (87%), often a close relative, neighbor, or school teacher. Faye Diemé et al.^18^ found that half of their patients (50.90%) knew their attackers. Pitché reports that in sub-Saharan Africa, 30–70% of assailants belong to the same family as the victim and are therefore known to her, which is not the case in regions where there is armed conflict^24^. The prevailing worldwide reports suggest that sexual assault by a stranger is uncommon^22,25,26^. This observation in our study may be explained by the unique context in which it was conducted (province), characterized by ongoing warfare, military violence, and recurring natural disasters that result in frequent population displacements. This context has resulted in the displacement of almost 5 million people and an estimated 16 million individuals in need of humanitarian assistance. The difference in our findings could be attributed to the fact that in our study setting, we are in a context of insecurity with almost 5 million people displaced and almost 16 million in need of humanitarian assistance, including a war in which SV has been used as a weapon^7,21^.

In the present study, more than 60.71% of the victims had been sexually raped under armed threat. In contrast, Kandolo et al.^16^ reported that the sexual aggressors in Lubumbashi, a town in the DRC that is not affected by armed conflict, were unarmed civilians. They also observed that military personnel, who have long been implicated in the DRC, especially during times of armed conflict, were poorly represented^16^. The proliferation of weapons among the population in the Eastern DRC, where armed conflict persists, contributes significantly to the high frequency of SV. Weapons, often used as instruments of power, exacerbate the vulnerability of women to armed aggressors. The widespread availability of weapons creates an environment conducive to abuse, and armed groups operating with impunity exploit this access to weapons to perpetrate SV^27^. This complex reality underlines the urgent need to address not only the consequences of attacks but also the root causes of the problem. Measures to reduce the circulation of weapons, combined with efforts to strengthen legal and judicial structures, are essential to reverse this alarming trend. Effective disarmament policies, combined with increased awareness of the devastating consequences of SV, could help create a safer environment for women in the province of North Kivu, where their fundamental rights are often neglected as a result of persistent conflict.

Based on the study’s findings, several key recommendations emerge for enhancing the case management of SV victims. There is an evident need for widespread dissemination of information regarding the availability and accessibility of medical and psychosocial services for SV survivors^28,29^. Furthermore, to encourage survivors to seek timely medical intervention, increased efforts in community-level awareness campaigns are necessary. These campaigns should aim to educate the public about the importance of prompt medical care, reduce stigma associated with SV, and promote the reporting of such incidents. In light of the high percentage of assaults by strangers, there is a clear imperative to bolster security and protective measures for women, especially in areas where displacement and instability are prevalent. This could involve community watch programs, better lighting in public spaces, and more proactive law enforcement presence. Moreover, there is a pressing need for legal reforms that address the widespread impunity for perpetrators of SV and strengthen the judicial response to these crimes, ensuring that survivors see justice served^15^. Training programs for healthcare professionals and community leaders on trauma-informed care and gender-sensitive approaches can further enhance the quality of services provided to survivors, fostering a culture of empathy, respect, and solidarity^30^.

Despite the insights gained from this study, it is important to acknowledge several limitations that may impact the interpretation and generalizability of the findings. Firstly, the study’s reliance on retrospective data from healthcare facilities may introduce biases, as not all cases of SV may be reported or documented. Data were collected from a limited number of health facilities—only four hospitals—which may not provide a comprehensive representation of the SV situation across the entire North Kivu province. Moreover, the scope of the study is limited to survivors who sought care at specific facilities in Goma, potentially excluding marginalized or hard-to-reach populations. The cross-sectional nature of the data also precludes the ability to establish causal relationships or track long-term outcomes for survivors. Additionally, the study’s focus on female survivors may overlook the experiences of male and gender non-conforming individuals, highlighting the need for more inclusive research approaches in future studies on conflict-related SV. The lack of reliable data on patient complaints and symptoms, insufficient data for certain variables such as specific places where SV occurs, are challenges that need to be addressed in future research.

Despite its limitations, this study contributes valuable insights to the understanding of SV and its impacts on survivors. The use of quantitative data from multiple healthcare facilities in Goma provides a comprehensive overview of the prevalence and patterns of SV in the region. By documenting the demographic characteristics, clinical presentations, and referral pathways of survivors, the study offers valuable information for improving the coordination and delivery of services for survivors of SV. Furthermore, the study’s focus on survivors’ perspectives and experiences adds depth to our understanding of the psychosocial and economic impacts of SV, highlighting the importance of holistic and survivor-centered approaches in case management and support services.

## Conclusion

This study provides valuable insights into SV among women in Goma. The majority of survivors were women aged 20–29 years old. A significant proportion of them sought post-sexual assault care within 72 h and received crucial services such as HIV testing and pregnancy examinations. Additionally, they were provided with contraception treatment and offered antiretroviral prophylactic medication. The assaults predominantly occurred outside the survivors’ homes and frequently involved the use of weapons. Notably, the perpetrators were often identified as strangers and civilians in most cases. Efforts should focus on raising awareness, providing timely support and care, addressing gender norms, and strengthening legal frameworks to combat SV and protect the rights of survivors. Future research should expand the scope, collaborate with more healthcare facilities, and improve data collection to enhance the understanding and response to this pressing public health issue.

## Data Availability

The data that support the findings of this study are available from the corresponding author upon reasonable request.

## Abbreviations

DRC: Democratic Republic of the Congo
GRH: General Referral Hospital
SV: Sexual violence
WHO: World Health Organization
